# Food and Beverage Availability in Small Food Stores Located in Healthy Food Financing Initiative Eligible Communities

**DOI:** 10.3390/ijerph14101242

**Published:** 2017-10-18

**Authors:** Chelsea R. Singleton, Yu Li, Ana Clara Duran, Shannon N. Zenk, Angela Odoms-Young, Lisa M. Powell

**Affiliations:** 1 Institute for Health Research and Policy, University of Illinois at Chicago, 1747 West Roosevelt Road, Chicago, IL 60608, USA; yli254@uic.edu; 2 Center for Food Studies, University of Campinas, Av. Albert Einstein, 291, Cidade Universitária, SP 13083-852, Brazil; anaduran@unicamp.br; 3 Department of Health Systems Science, College of Nursing, University of Illinois at Chicago, 845 South Damen Avenue, Office 960, Chicago, IL 60608, USA; szenk@uic.edu; 4 Department of Kinesiology and Nutrition, College of Applied Health Sciences, University of Illinois at Chicago, 1919 West Taylor Street, Office 709, Chicago, IL 60608, USA; odmyoung@uic.edu; 5 Division of Health Policy and Administration, University of Illinois at Chicago, 1747 West Roosevelt Road, Office 448, Chicago, IL 60608, USA; powelll@uic.edu

**Keywords:** Healthy Food Financing Initiative, food desert, grocery store, convenience store, low-income, African-American, Illinois

## Abstract

Food deserts are a major public health concern. This study aimed to assess food and beverage availability in four underserved communities eligible to receive funding from the Healthy Food Financing Initiative (HFFI). Data analyzed are part of a quasi-experimental study evaluating the impact of the HFFI on the retail food environment in selected Illinois communities. In 2015, 127 small grocery and limited service stores located in the four selected communities were audited. All communities had a large percentage of low-income and African-American residents. Differences in food and beverage item availability (e.g., produce, milk, bread, snack foods) were examined by store type and community location. Food stores had, on average, 1.8 fresh fruit and 2.9 fresh vegetable options. About 12% of stores sold low-fat milk while 86% sold whole milk. Only 12% of stores offered 100% whole wheat bread compared to 84% of stores offering white bread. Almost all (97%) stores offered soda and/or fruit juice. In summary, we found limited availability of healthier food and beverage items in the communities identified for HFFI support. Follow up findings will address how the introduction of new HFFI-supported supermarkets will affect food and beverage availability in these communities over time.

## 1. Introduction

Disparities in the availability of food retailers that offer healthy affordable food items have been documented in the U.S. [1,2,3,4,5,6]. Compared to majority affluent and white communities, majority low-income and African American communities often have reduced access to healthy food retailers such as supermarkets [1,2,4]. The United States Department of Agriculture (USDA) estimates that nearly 50% of the 23.5 million people who reside in communities they recognize as food deserts are low-income [7,8]. Food access disparities have been linked to public health concerns such as poor dietary intake and, in some cases, obesity [9,10,11,12,13]. Considering low-income and African American populations are disproportionately impacted by obesity and chronic diseases such as diabetes and hypertension [14,15], the retail food environment may play a considerable role in the development of racial and socioeconomic disparities in health [16,17].

The scientific literature further highlights issues surrounding the healthfulness of foods and beverages sold by food stores in underserved communities [18,19,20,21,22,23,24,25,26]. Previous research has found that food stores in underserved communities often carry few fresh fruit and vegetable options and a large supply of calorically-dense foods, snacks, and sugar-sweetened beverages [5,20,24,25]. This issue is further exacerbated by the fact that underserved communities in urban centers often have a large number of small food stores (e.g., convenience stores, liquor stores) and no supermarket [26,27,28].

Much national attention has been given to policies and initiatives that aim to increase the amount of healthy foods offered by food retailers in underserved communities [29,30,31,32,33,34,35,36]. The Healthy Food Financing Initiative (HFFI) is a federal initiative launched by the Obama Administration in 2011 to increase healthy food retail in underserved communities throughout the U.S. [37,38,39]. The HFFI is one of the largest federal initiatives in U.S. history to address healthy food access disparities [37,38,39]; since its implementation, the HFFI has supported the expansion of healthy foods being sold in communities identified by the USDA as being food deserts [38]. Several communities in the U.S. have been deemed HFFI-eligible, and some have since been able to address their ongoing need for healthy food because of HFFI funding. 

Organizations such as the Illinois Fresh Food Fund and the Chicago Community Loan Fund received HFFI funding to support the development of new chain supermarkets in HFFI-eligible communities in Rockford, IL, USA, Chicago, IL, USA and suburban areas surrounding Chicago. The communities where the HFFI-supported supermarkets were developed had high percentages of low-income and African American residents and a high density of small food stores present in the area prior to the supermarket openings. 

The objective of this research was to assess the availability of foods and beverages in four HFFI-eligible Illinois communities. At the time of data collection, two of these communities were developing a new HFFI-supported supermarket. We collected information on food and beverage availability prior to the supermarket openings. By assessing the food and beverage availability in these HFFI-eligible communities, we can (1) better characterize the need for the HFFI-supported supermarkets; (2) further examine the role of food store type on the supply of staple food items in underserved communities; (3) document the availability of healthy foods in stores authorized to accept Supplemental Nutrition Assistance Program (SNAP) benefits; and (4) provide the scientific literature updated information about the ongoing issue of food deserts in the U.S. Overall, this research sheds light on the existing food landscape of communities identified by this large-scale federal initiative as having poor access to healthy foods.

## 2. Materials and Methods

### 2.1. Selected Communities

To accomplish our research objective, we analyzed the baseline data collected from a 5-year quasi-experimental study being conducted by the Illinois Prevention Research Center (IPRC) and the Nutrition and Obesity Policy Research Evaluation Network (NOPREN) Collaborating Center at the University of Illinois at Chicago (UIC). The overarching goal of the IPRC-NOPREN project is to examine longitudinal changes to the retail food environment of HFFI-eligible communities that are receiving a large HFFI-supported chain supermarket. Two communities were selected for inclusion in the IPRC-NOPREN project. Inclusion criteria included (1) the nearest supermarket was more than one mile from the identified HFFI-supported supermarket site and (2) the HFFI-supported supermarket was scheduled to open after 1 August 2015, which fit the IPRC-NOPREN project’s timeline.

The first community selected was located on Chicago’s south side. With HFFI funds, the Chicago Community Loan Fund and a private realty group developed a Whole Foods Market, which opened for business in October 2016. Approximately 98% of the people residing within a one-mile radius around the Whole Foods location are African American and 44% live below the federal poverty line. The second community selected was west Rockford, a neighborhood in Rockford, IL, USA. Rockford, IL, USA is a small city located about 90 miles northwest of Chicago. The Illinois Fresh Food Fund developed a Save-A-Lot supermarket in west Rockford, which opened for business at the end of August 2015. In west Rockford, 48% of the residents living within a one-mile radius of the Save-A-Lot are African American and 47% live below the federal poverty line.

A demographically-matched HFFI-eligible comparison community was selected for each intervention site. The two comparison communities were selected because they had similar percentages of African American and impoverished residents, no supermarket available, and no HFFI-supported project in development. Comparison communities were located on Chicago’s west side and in Rockford (south of the intervention community). The Institutional Review Board at UIC considered this research exempt.

### 2.2. Data Collection and Food Store Observation Tool

A complete audit of all existing food stores (e.g., convenience stores, liquor stores, pharmacies, dollar stores) located in the two intervention communities and the two comparison communities was conducted to collect key data on food and beverage availability. Baseline data were collected in 2015 prior to the opening of the HFFI-supported supermarkets. For HFFI communities, a complete audit of the food retail stores located within a one-mile buffer of the HFFI-funded supermarket’s address was conducted. For comparison communities, a complete audit of food retail stores located within a one-square mile area was conducted. All stores that sold food (i.e., small grocery stores, convenience stores, drug stores/pharmacies, liquor stores, discount stores, dollar stores, etc.) were included in the audit. Fast food restaurants and full service restaurants were not examined.

Data were collected using the IPRC-NOPREN Food Store Observation Form which was adapted from the validated Bridging the Gap Food Store Observation Form (BTG-FSOF) [40]. Details on the development, validation, inter-rater reliability, and pilot testing of the BTG-FSOF were previously described in 2013 [40]. The IPRC-NOPREN Food Store Observation Form features over 600 items designed to objectively assess food and beverage availability and general store features. Field workers were trained for one week to use the food store observation form and employed to conduct the baseline audits of eligible small food stores in the four study communities. Baseline data were collected in August and September 2015.

### 2.3. Measures

#### 2.3.1. General Store Features

Information on food store type, interior store features, and exterior store features was collected from every store audited. Field workers recorded the store type as either small grocery store or limited service store. Outlets qualified as food stores if they sold at least five different food items being assessed on the IPRC-NOPREN Food Store Observation Form. Stores were classified as small grocery stores if they sold fresh meat. Otherwise, there were considered a limited service store. Field workers further classified the limited service store’s type as convenience store, small discount store, drug store/pharmacy, or liquor store. Convenience stores sold convenience items (i.e., ready-to-eat or ready-to-heat foods) and offered a limited line of groceries (e.g., 7-Eleven). Stores at gas stations were also coded as convenience stores. Small discount stores were stores that offered a general line of groceries as well as non-food items such as clothing and household cleaning products (e.g., Dollar General, Dollar Tree). Drug stores and pharmacies sold prescription medication in addition to food and beverage items (e.g., Walgreens, CVS). Alcohol products had to make up at least an estimated 50% of the store inventory for it to be considered a liquor store (e.g., Binny’s Beverage Depot).

Field workers also collected information on the number of cash registers, SNAP authorization status, and service counter availability. Field workers searched for signage inside and outside the store that indicated the establishment was authorized to accept SNAP benefits. If no signage was found, they asked a store clerk or manager about the acceptance of SNAP. Field workers recorded (yes or no) if the store had a butcher, bakery, or deli service counter available. Furthermore, field workers recorded (yes or no) if the store sold gasoline or had parking available on site.

#### 2.3.2. Food and Beverage Availability

Availability was assessed for a wide range of food and beverage items. The IPRC-NOPREN Food Store Observation Form permits the individual assessment of 20 different fresh fruit and vegetable items that are commonly consumed in the U.S. or culturally relevant to African Americans [41]. These items include tomatoes, lettuce, bell peppers, bananas, apples, oranges, grapes, watermelons, cabbages, sweet potatoes, celery, carrots, greens, broccoli, avocados, corn, spinach, okra, chard, and beets. Field workers recorded (yes or no) if the fresh fruit and vegetable item was available in the store. Furthermore, study staff recorded the following counts: total number of fresh fruit options (top coded at 20+), total number of fresh vegetable options (top coded at 20+), total number of frozen fruit options (top coded at 10+), total number of frozen vegetable options (top coded at 10+), total number of canned/shelf stable fruit options (top coded at 10+), and total number of canned/shelf stable vegetable options (top coded at 10+).

The availability of regular versus healthier alternatives for a variety of food items was assessed: bread (white vs. 100% whole wheat), cereal (high-sugar vs. low-sugar), rice (white vs. brown), cheese (whole-fat vs. reduced-fat), plain yogurt (whole-fat vs. reduced-fat), ground beef (regular vs. lean) and potato chips (regular vs. low-fat). Field workers were trained to thoroughly assess the packaging of food items to identify that a food item met specific study definitions. Bread packaging had to explicitly specify that it was “100% whole wheat” in order for the store to be considered as having 100% whole wheat bread. Cereal with <6 g of sugar per serving was considered a low-sugar cereal. Packaging for cheese had to say “reduced-fat” or “part-skim” to be considered reduced-fat. Packaging for plain yogurt had to say “reduced-fat”, “low-fat”, or “non-fat” to be considered reduced-fat. Ground beef with ≤10% fat was considered lean ground beef. Potato chips with <4 g of fat per 1-ounce serving were considered low-fat potato chips.

The availability of regular versus healthier alternatives for a variety of beverage items was assessed: milk (regular vs. reduced-fat), juice (<50% or fruit punch vs. 100% juice.), soda (regular vs. diet), and water (enhanced vs. plain). Skim and 1% milk were considered reduced-fat milk, while whole and 2% milk were considered regular milk. Only unflavored cow’s milk was considered for these items. Packaging for juice had to explicitly state that the item was 100% juice in order for it to be considered 100% juice. Soda packaging had to say “diet” or “zero-calorie” for the item to be considered diet soda. Beverage items advertised as water but containing other items such as sugar, sweeteners, artificial flavoring, vitamins, or minerals were considered enhanced water. Examples given to study staff included Vitamin Water and Sobe Life Water.

### 2.4. Statistical Analysis

Baseline data collected by the field workers were entered into a password secure REDCap database and checked for completion by study staff. A detailed logic checking protocol was applied to the data to ensure data quality prior to performing statistical analyses. Analyses were performed by STATA version 14 (STATA—College Station, TX, USA). Descriptive statistics (i.e., means and frequencies) were calculated for measures of availability among all food stores (N = 127) and all SNAP-authorized food stores (N = 108) and were stratified by food store type (small grocery store vs. limited service store). Chi-square statistics were used to examine differences in frequencies. Student’s *t* tests were used to examine differences in means. *p* values less than 0.05 were considered statistically significant.

## 3. Results

### 3.1. Store Features

Interior and exterior features of food stores are presented in Table 1 for all stores and SNAP authorized stores and results are stratified by store type. At baseline, 152 eligible stores were found in the four communities examined. Field workers were unable to audit 25 of these stores because the food store was 100% clerk-assisted (N = 18), the store staff asked them to leave before completing the data collection (N = 6), or the store location was not safe (N = 1). This resulted in a final sample size of 127 stores (40 located in Rockford and 87 located in Chicago). There were 34 (27%) small grocery stores and 93 (73%) limited service stores. Of the 93 limited service stores, 72% of them were convenience stores. The remaining 28% were pharmacies, liquor stores, discount stores, or dollar stores. Mean number of cash registers among all stores was 1.6 (±0.9). The vast majority of stores accepted SNAP benefits (108 of 127). All small grocery stores accepted SNAP benefits compared to 81% of limited service stores (*p* = 0.01). 

### 3.2. Fruit and Vegetable Availability

**Figure 1:**
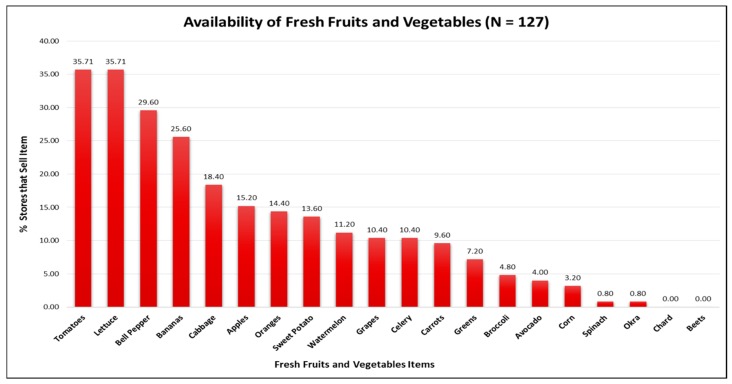
Fresh fruit and vegetable item availability in food stores across all sites.

Figure 1 displays availability information among all 127 food stores for the 20 fresh fruit and vegetable items included in the store audit. Red tomatoes (35.7%), lettuce (35.7%), and bell peppers (29.6%) were fresh produce items observed the most. A lower percentage of stores carried bananas (29.6%) and apples (15.2%). No stores offered fresh chard or beets. Table 2 shows that, on average, food stores offered more canned and shelf-stable fruit and vegetable options compared to fresh and frozen fruit and vegetable options. Limited service stores and grocery stores differed significantly with respect to the mean number of available fresh, frozen, and canned/shelf-stable fruit and vegetable items. On average, limited service stores had a significantly lower number of fresh fruit options (0.7 vs. 4.8; *p* < 0.0001), fresh vegetable options (0.8 vs. 8.8; *p* < 0.0001), frozen vegetable options (1.0 vs. 4.8; *p* < 0.0001), canned/shelf-stable fruit options (3.2 vs. 5.1; *p* < 0.0001), and canned/shelf-stable vegetable options (6.1 vs. 8.7; *p* < 0.0001) compared to grocery stores. The mean number of fruit and vegetable options in the SNAP-authorized stores was similar to the overall mean.

### 3.3. Food and Beverage Availability

Table 3 displays information on food and beverage item availability for all food stores and SNAP authorized food stores and results are stratified by store type. Approximately 12.1% of stores offered 100% whole wheat bread while 84.0% of stores offered only white bread. High-sugar cereal was more prevalent at 88.8% compared to low-sugar cereal at 55.2%. White rice was available in 88.8% of all food stores while brown rice was available in 21.0% of stores. Regular-fat cheese was offered in 46.5% of all stores assessed, whereas reduced-fat cheese was offered in 31.8% of all stores. About 6.3% of all stores had lean ground beef (i.e., ≤10% fat), whereas 18.1% of all stores had regular ground beef (i.e., ≥20% fat). Although 9.0% of all stores had baked or low-fat chips, 96.0% of all food stores examined had regular-fat plain potato chips. Whole and/or 2% reduced-fat milk was offered by 85.8% of stores, yet 1% reduced-fat and/or skim milk was offered by 11.8% of stores. Fruit punch and juice drinks (<50% juice) were available in 96.8% of all examined stores and 100% fruit juice was available in 88.7% of all stores. Approximately 97.6% of all stores carried regular soda, and 76.6% carried diet soda. Plain bottled water was available in 98.4% of all food stores, and enhanced water was available in 42.7% of all food stores. Food and beverage availability in SNAP-authorized stores was similar to the total sample of stores.

Significant differences were observed between small grocery stores and limited service stores with respect to the availability of brown rice, regular-fat cheese, reduced-fat cheese, diet soda, and enhanced water. A significantly higher percentage of small grocery stores sold brown rice (33.3% vs. 16.5%; *p* = 0.04), reduced-fat cheese (60.6% vs. 21.5%; *p* < 0.0001), and regular-fat cheese (82.4% vs. 33.3%; *p* < 0.0001) compared to limited service stores. A higher percentage of limited service stores offered enhanced water compared to small grocery stores (48.4% vs. 27.3%; *p* = 0.04).

## 4. Discussion

This research aimed to examine food and beverage availability in four HFFI-eligible Illinois communities; two of which recently received a new HFFI-supported chain supermarket. To accomplish this aim, we examined the baseline data collected by the IPRC-NOPREN project prior to the supermarket openings. At baseline, we found 127 small grocery and limited service stores in the two selected communities and their demographically-matched comparison communities. Eighteen stores were located within a 1-mile radius of the Save-A-Lot location in west Rockford and 47 were located within a 1 mile radius of the Whole Foods Market location in Chicago. Despite the large number of food retailers located in the intervention communities, the availability of staple food and beverage items often promoted as being healthy (e.g., fruits and vegetables, 100% whole wheat bread, low-fat milk, etc.) was low. These findings not only mirror the results from other studies of food and beverage availability in underserved communities [18,19,20,21,22,23,24,25,26,27,28], they underscore each community’s need for a large grocer such as the HFFI-supported supermarket.

Similarities and differences in the availability of specific food items were observed between small grocery stores and limited service stores. For example, there was no difference between small grocery stores and limited service stores with respect to the percentage of stores that carried 100% whole wheat bread and low-fat milk (i.e., 1% and skim milk). The availability of both items was low among the two food store types, which suggest that being designated a “grocery store” may not always indicate higher availability of healthier food options. Only 9% of the limited service stores and 21% of the small grocery stores audited carried 100% whole wheat bread. Furthermore, low-fat milk was stocked in only 12% of all stores. Liese and colleagues (2007) also reported low availability of high fiber bread in a sample of 57 convenience stores in South Carolina [28]. Zenk and colleagues reported in their 2010 study that 15% of 157 food stores they audited in southwest Chicago carried low-fat milk [22]. Disparities in milk availability have been documented at the local and national levels [20,23,28]. In a nationwide examination of milk availability using data from 468 communities, Rimkus and colleagues (2015) observed that the odds of a food store carrying low-fat milk were up to 44% lower in low-income communities [23], and the odds of a food store carrying any type of milk were up to 67% lower in African American communities [23].

The key difference between the small grocery stores and limited service stores was the stocking of fruits and vegetables. For example, limited service stores carried significantly fewer options for fruits and vegetables compared to small grocery stores. Limited service stores averaged less than one option for fresh and frozen fruits and vegetables. Canned and shelf-stable fruits and vegetables were more prevalent in both types of stores. Recent studies by Caspi and colleagues (2016) and Laska and colleagues (2015) found a similar pattern of fruit and vegetable availability in small food stores located in Minneapolis and St. Paul, MN, USA [21,27]. In the current study, we assessed individual fruit and vegetable items to determine if certain items previously shown to be more culturally relevant to African Americans (e.g., sweet potatoes, collard greens, okra, etc.) were prevalent in small food stores. Lettuce, tomatoes, and bell peppers were the most available fresh produce items despite less than 40% of all stores we audited having these items in stock. A study by Grigsby-Toussaint and colleagues (2010) previously reported that African American neighborhoods in Chicago, including Englewood, were likely to sell culturally relevant fruits and vegetables; however, less than 50% of the stores they assessed carried any fresh fruits and vegetables [42].

As expected, the availability of calorically dense snack foods and sugar-sweetened beverages (SSBs) was high in the small food stores included in our sample. Chips, soda, and drinks containing <50% juice were found is almost every store. The availability of fruit punch and beverages containing <50% juice was higher than the availability of 100% fruit juice. The availability of low-fat chips (<4 g of fat per 1 ounce serving) was low in both small grocery and limited service stores. Previous research suggests that snack food and sugar-sweetened beverage availability in small food stores can vary greatly between geographic areas [18,25]. Income level and racial composition of the surrounding community are believed to be key factors that influence the stocking practices of snack foods and beverage items in small food stores [25]. Additional research is needed on the stocking practices of healthier beverages and snack items among food retailers in underserved communities and on how the introduction of a large and/or chain grocer influences these stocking practices over time.

It is important to note that 85% of the small food stores we audited were authorized to accept SNAP benefits. Considering the HFFI-eligible communities selected by the IPRC-NOPREN project previously lacked a supermarket, it is probable that the community residents participating in SNAP who lack sufficient transportation relied, in part, on nearby small food stores to purchase staple food items. The limited availability of healthy staple food items reduces the autonomy community residents possess to make healthy food purchasing decisions for their families. This highlights an important and on-going topic of discussion related to the amount of healthy foods SNAP-authorized food retailers are required to stock [41]. Researchers, policy makers, and key stakeholders examining this issue are concerned about the stocking practices of SNAP-authorized stores, and changes to the stocking requirements have been proposed [41]. The results from this study show that the overwhelming majority of the small food stores examined are in fact SNAP-authorized retailers, and despite this their offerings of healthy foods were quite limited. Future work should study how stronger stocking requirements for SNAP authorization status may increase staple food offerings in small stores. 

The strengths and limitations of this research should be noted. The data source was a strength because it included detailed food and beverage availability information on a full audit of small food retail stores located in two communities identified by a federal initiative as being food deserts. Use of the validated IPRC-NOPREN Food Store Observation Form to perform the food store audits was also a strength because it allowed the assessment of over 600 items including a wide variety of beverages, staple food items, and culturally-specific fruits and vegetables. A key limitation to this research is the small sample size of food retail stores. It is likely that the low number of stores included in the sample affected our ability to observe differences in food and beverage availability between grocery stores and limited services stores. Furthermore, considering this project involves HFFI-eligible communities in Illinois only, findings from this research study may not be generalizable to underserved communities in other geographic areas.

## 5. Conclusions

In summary, the baseline data from the IPRC-NOPREN project revealed that the availability of healthy staple food and beverage items was low in the small grocery and limited service stores found in selected HFFI-eligible communities in Illinois. This finding demonstrates the need for a HFFI-supported chain or large supermarket even in underserved communities that already have grocery stores and a high density of SNAP-authorized food retail outlets. It also provides evidence to the scientific literature that suggests the issue of food deserts persists in the U.S. In recent years, researchers have started evaluating various impacts of the HFFI [43,44,45,46,47]; however, additional information is needed in the literature. Future findings from the IPRC-NOPREN project will describe the longitudinal changes to food availability, pricing, and marketing in the existing small food stores after the opening of new HFFI-supported chain supermarkets in Chicago and Rockford, IL, USA. We hypothesize that the new supermarkets will improve the availability of staple food items in these communities and influence the business and stocking practices of the nearby existing small food stores.

## Figures and Tables

**Table 1:** Features of small food stores stratified by store type, % or mean (SD).

Store Feature	All Stores N = 127	Small Grocery N = 34	Limited Service N = 93	*p*-Value ^b^	SNAP Authorized N = 108
Limited Service Store Type:					
Convenience Store	52.8%	-	72.0%	-	50.9%
Other **^a^**	20.5%	-	28.0%	-	18.5%
Number of Cash Registers	1.6 (0.9)	1.7 (1.0)	1.6 (0.9)	0.64	1.7 (0.9)
Accepts SNAP Benefits	86.4%	100.0%	81.5%	0.008	100%
Butcher, Bakery, or Deli Available	26.2%	75.8%	8.6%	<0.0001	28.7%
Parking On-Site Available	54.4%	43.8%	58.1%	0.16	52.3%

HFFI, Healthy Food Financing Initiative; SNAP, Supplemental Nutrition Assistance Program; **^a^** Other limited service stores include pharmacies, liquor stores, and discount/dollar stores; **^b^** *p* value examining difference between small grocery stores and limited service stores.

**Table 2:** Fruit and vegetable availability in food stores stratified by store type, mean (SD).

Presentation	All Stores N = 127	Small Grocery N = 34	Limited Service N = 93	*p*-Value ^a^	SNAP Authorized N = 108
Fresh:					
Number of Fruit Options	1.8 (4.0)	4.8 (6.0)	0.7 (2.2)	<0.0001	1.7 (3.7)
Number of Vegetable Options	2.9 (4.6)	8.8 (5.2)	0.8 (1.7)	<0.0001	2.7 (4.8)
Frozen:					
Number of Fruit Options	0.2 (0.9)	0.6 (1.6)	0 (0.0)	0.0003	0.1 (0.6)
Number of Vegetable Options	2.0 (2.8)	4.8 (3.2)	1.0 (1.8)	<0.0001	1.9 (3.0)
Canned and Shelf-Stable:					
Number of Fruit Options	3.7 (2.3)	5.1 (2.5)	3.2 (2.1)	<0.0001	3.8 (2.4)
Number of Vegetable Options	6.8 (3.3)	8.7 (2.5)	6.1 (3.2)	<0.0001	6.8 (3.1)

HFFI, Healthy Food Financing Initiative; **^a^** *p* value examines difference between small grocery stores and limited service stores.

**Table 3:** Food and beverage availability in food stores stratified by store type, %.

Item	All Stores N = 127	Small Grocery N = 34	Limited Service N = 93	*p*-Value ^a^	SNAP Authorized N = 108
Bread:					
100% Whole Wheat Bread	12.1%	21.2%	8.8%	0.06	14.2%
White Bread	84.0%	93.9%	80.4%	0.07	90.7%
Cereal:					
Low-Sugar (<6 g)	55.2%	66.7%	51.1%	0.12	59.8%
High-Sugar (≥6 g)	88.8%	90.9%	88.0%	0.28	94.4%
Rice:					
Brown Rice	21.0%	33.3%	16.5%	0.04	23.6%
White Rice	88.8%	93.9%	87.0%	0.28	90.7%
Cheese:					
Low or Reduced-Fat	31.8%	60.6%	21.5%	<0.0001	35.2%
Regular-Fat	46.5%	82.4%	33.3%	<0.0001	50.9%
Yogurt:					
Low-Fat or Non-Fat	4.8%	8.8%	3.3%	0.19	4.7%
Whole-Fat or Regular	1.6%	2.9%	1.1%	0.46	0.9%
Ground Beef:					
Extra Lean (≤10% Fat)	6.3%	23.5%	0.0%	<0.0001	7.4%
Regular (≥20% Fat)	18.1%	67.7%	0.0%	<0.0001	20.4%
Potato Chips (Plain):					
Baked or Low-Fat	9.0%	9.1%	9.0%	0.99	10.4%
Regular	96.0%	90.9%	97.9%	0.08	96.3%
Milk:					
1% or Skim	11.8%	14.7%	10.8%	0.54	12.0%
Whole or 2%	85.8%	94.1%	82.8%	0.11	92.6%
Juice:					
100% Juice	88.7%	84.9%	90.1%	0.41	89.6%
<50% Juice or Fruit Punch	96.8%	97.0%	96.7%	0.94	97.2%
Soda:					
Diet	76.6%	63.6%	81.3%	0.04	75.5%
Regular	97.6%	97.0%	97.8%	0.94	98.1%
Water:					
Plain	98.8%	97.0%	98.9%	0.45	99.1%
Enhanced	42.7%	27.3%	48.4%	0.04	43.4%

HFFI, Healthy Food Financing Initiative; **^a^** *p* value examines difference between small grocery stores and limited service stores.
